# A Non-Hemadsorbing Live-Attenuated Virus Vaccine Candidate Protects Pigs against the Contemporary Pandemic Genotype II African Swine Fever Virus

**DOI:** 10.3390/v16081326

**Published:** 2024-08-19

**Authors:** Quang Lam Truong, Lihua Wang, Tuan Anh Nguyen, Hoa Thi Nguyen, Anh Dao Le, Giap Van Nguyen, Anh Thi Vu, Phuong Thi Hoang, Trang Thi Le, Huyen Thi Nguyen, Hang Thu Thi Nguyen, Huong Lan Thi Lai, Dao Anh Tran Bui, Le My Thi Huynh, Rachel Madera, Yuzhen Li, Jamie Retallick, Franco Matias-Ferreyra, Lan Thi Nguyen, Jishu Shi

**Affiliations:** 1 Key Laboratory of Veterinary Biotechnology, Faculty of Veterinary Medicine, Vietnam National University of Agriculture, Gia Lam, Ha Noi 12406, Vietnam; anhanhtuan997@gmail.com (T.A.N.); hoanguyen2405@gmail.com (H.T.N.); daoleanhvetlab@gmail.com (A.D.L.); vuanhhd98@gmail.com (A.T.V.); htphuong1989@gmail.com (P.T.H.); lttranglaboratory@gmail.com (T.T.L.); nguyenhuyen@vnua.edu.vn (H.T.N.); hangthu780@gmail.com (H.T.T.N.); ltlhuong@vnua.edu.vn (H.L.T.L.); btadao@vnua.edu.vn (D.A.T.B.); 2 Center on Biologics Development and Evaluation, Department of Anatomy and Physiology, College of Veterinary Medicine, Kansas State University, Manhattan, KS 66506, USA; lihua@vet.k-state.edu (L.W.); rachelmadera@vet.k-state.edu (R.M.); yuzhen@vet.k-state.edu (Y.L.); 3 Department of Veterinary Microbiology and Infectious Diseases, Faculty of Veterinary Medicine, Vietnam National University of Agriculture, Gia Lam, Ha Noi 12406, Vietnam; nvgiap@vnua.edu.vn (G.V.N.); huynhtmle@yahoo.com (L.M.T.H.); 4 Department of Diagnostic Medicine and Pathobiology, College of Veterinary Medicine, Kansas State University, Manhattan, KS 66506, USA; retallick@vet.k-state.edu (J.R.); francomf@vet.k-state.edu (F.M.-F.)

**Keywords:** African swine fever, live-attenuated virus, safe, efficacious, genotype II, protection

## Abstract

African swine fever (ASF) is a highly contagious and severe hemorrhagic transboundary swine viral disease with up to a 100% mortality rate, which leads to a tremendous socio-economic loss worldwide. The lack of safe and efficacious ASF vaccines is the greatest challenge in the prevention and control of ASF. In this study, we generated a safe and effective live-attenuated virus (LAV) vaccine candidate VNUA-ASFV-LAVL3 by serially passaging a virulent genotype II strain (VNUA-ASFV-L2) in an immortalized porcine alveolar macrophage cell line (3D4/21, 50 passages). VNUA-ASFV-LAVL3 lost its hemadsorption ability but maintained comparable growth kinetics in 3D4/21 cells to that of the parental strain. Notably, it exhibited significant attenuation of virulence in pigs across different doses (10^3^, 10^4^, and 10^5^ TCID_50_). All vaccinated pigs remained healthy with no clinical signs of African swine fever virus (ASFV) infection throughout the 28-day observation period of immunization. VNUA-ASFV-LAVL3 was efficiently cleared from the blood at 14–17 days post-infection, even at the highest dose (10^5^ TCID_50_). Importantly, the attenuation observed in vivo did not compromise the ability of VNUA-ASFV-LAVL3 to induce protective immunity. Vaccination with VNUA-ASFV-LAVL3 elicited robust humoral and cellular immune responses in pigs, achieving 100% protection against a lethal wild-type ASFV (genotype II) challenge at all tested doses (10^3^, 10^4^, and 10^5^ TCID_50_). Furthermore, a single vaccination (10^4^ TCID_50_) provided protection for up to 2 months. These findings suggest that VNUA-ASFV-LAVL3 can be utilized as a promising safe and efficacious LAV candidate against the contemporary pandemic genotype II ASFV.

## 1. Introduction

African swine fever (ASF) is a lethal and highly contagious transboundary animal disease with the potential for rapid international spread [1]. The clinical signs and gross pathological lesions of ASF in swine may vary depending on the virus isolate, infection route, dose, and host characteristics. Acute ASF presents with high fever (up to 42 °C), lethargy, anorexia, and inactivity [2]. The causative agent, African swine fever virus (ASFV), is a large, enveloped, double-stranded DNA virus belonging to the *Asfivirus* genus within the *Asfarviridae* family. Its genome is approximately 170–194 kilobase pairs (kb) and contains over 150 open reading frames (ORFs), depending on the virus strain [3]. Based on the p72 major capsid protein gene (*B646L*), 24 ASFV genotypes (I–XXIV) have been identified [4]. 

ASF was first reported in Africa in 1921 and emerged for the first time in Europe in I957 [3,4]. In 2007, a highly pathogenic strain emerged in the Caucasus region of the Republic of Georgia and swiftly spread to neighboring countries such as the Russian Federation, Armenia, Ukraine, and Azerbaijan [5,6]. From 2014 to 2018, ASFV re-emerged in the European Union, originating from eastern nations to Lithuania, Poland, Latvia, and Estonia, subsequently spreading to Hungary, the Czech Republic, and Romania [7,8]. The situation escalated globally when ASF reached China in 2018 and spread swiftly across Asia, including Vietnam [9,10,11,12,13]. In 2021, it re-emerged in the Western Hemisphere on the Caribbean Island of Hispaniola (Dominican Republic and Haiti) after nearly 40 years [14,15]. ASF has also spread westward across Europe in recent years. After a 40-year absence, the disease re-emerged in Italy in January 2022 [16]. The outbreak has continued to spread within the country. In 2023, ASF was confirmed for the first time on domestic pig farms in Bosnia and Herzegovina, Greece, and Croatia [17]. The disease has now been reported on every continent except Antarctica, despite control efforts in some regions [6,18,19].

The absence of a safe and effective vaccine remains a critical hurdle in controlling ASF. Despite substantial research efforts in recent years, developing a protective ASF vaccine has proven challenging. While inactivated vaccines are safe, their efficacy in preventing infection in pigs is limited, as demonstrated by numerous clinical trials evaluating various ASFV strains, inactivation methods, adjuvants, and vaccination regimens [18,19,20,21]. Subunit vaccines, targeting individual or multiple ASF antigens, have also shown insufficient protection [18,21,22]. Live-attenuated vaccines (LAVs) have shown greater promise, offering improved immune protection compared to inactivated and subunit approaches [23]. These vaccines reduce virulence through natural passage or artificial cell passage or genetic modification, while preserving immunogenicity. Early attempts in the 1960s focused on adapting and attenuating ASFV through passaging in primary cell cultures, including bone marrow (BM), buffy coat (BC), pig leukocyte (PL), primary pig kidney cells (PPKs), primary bone marrow cells (PBMCs), and porcine alveolar macrophages (PAMs) [24,25]. However, the time-consuming and costly process of obtaining primary cells, coupled with ethical concerns, hindered large-scale production and research. To overcome these limitations, researchers successfully adapted ASFV strains to continuous cell lines such as COS-1, CV1, IPAM, MA104, WSL, PIPEC, A4C2/9K, ZMAC-4, IPKM, PPK-66b, and 3D4/21 [24,25,26]. This shift towards continuous cell lines has significantly facilitated ASFV research and vaccine development. Since the ASF outbreak, researchers worldwide have developed several LAVs, such as cell-adapted strains (L’60BM89, ASFV-G/VP110, and VNUA-ASFV-LAVL2) and naturally attenuated isolates (NH/P68 and Lv17/WB/Rie1) [27,28,29,30,31]. 

Advancements in ASFV genomics have facilitated the creation of gene-deleted LAV candidates. These LAVs achieve attenuation by removing specific virulence genes while maintaining protective immunity. Examples include single-gene deletions (ASFV-G-∆I177L, ASFV-G-ΔA137R, and SY18ΔI226R) and multiple-gene deletions (HLJ/18-7GD, ASFV-G-ΔMGF, ASFV-GΔ9GL/ΔCD2v, and ASFV-G-ΔI177LΔLVR) [32,33,34,35,36,37,38]. While the seven-gene deletion and *I177L* gene deletion strains have progressed to clinical trials in China and Vietnam [32,38], respectively, commercialization of ideal LAVs faces challenges related to large-scale production, vaccine stability, safety, and the development of companion diagnostics for differentiating infected from vaccinated animals (DIVA) [21,28,29].

Responding to the ASF pandemic, our research group has intensified efforts towards ASF vaccine development since 2019 [18,26]. Here, we report the generation of a non-hemadsorbing LAV candidate from a virulent genotype II ASFV using cell passage, and the evaluation of its safety and efficacy in pigs. 

## 2. Materials and Methods

### 2.1. Cells and Viruses 

Primary pulmonary alveolar macrophages (PAMs) were prepared as previously described [26,39] and maintained in a medium containing Dulbecco’s modified Eagle medium (DMEM, Life Technologies, Grand Island, NY, USA) supplemented with 10% heat-inactivated fetal bovine serum (FBS, Thermo Scientific, Waltham, MA, USA) and 1% antimycotic (Life Technologies, Grand Island, NY, USA) at 37 °C in a 5% CO_2_ incubator.

3D4/21 (immortalized porcine alveolar macrophage cell line, ATCC, CRL-2843) cells were cultured in an RPMI 1640 medium (Life Technologies, Grand Island, NY, USA) supplemented with 10% heat-inactivated fetal bovine serum (FBS, Gibco, Thermo Scientific, Waltham, MA, USA), 1% antimycotic (Life Technologies, Grand Island, NY, USA), and 1% MEM Non-Essential Amino Acids Solution (Thermo Scientific, Waltham, MA, USA) at 37 °C in a 5% CO_2_ incubator. 

The wild-type VNUA-ASFV-05L1 strain (genotype II) was isolated from the spleen of a domestic pig with typical acute ASF during an ASF outbreak in Northern Vietnam in 2020 [40]. The virulent VNUA-ASFV-L2 strain was generated by serially passaging wild-type VNUA-ASFV-05L1 in PAMs (70 passages). The live-attenuated vaccine candidate VNUA-ASFV-LAVL3 was generated by serially passaging VNUA-ASFV-L2 in 3D4/21 cells (50 passages). All viruses were maintained in BSL-3 laboratories of Vietnam National University and Kansas State University. 

### 2.2. Animals 

All animal care and protocols were reviewed and approved by the Institutional Animal Care and Use Committee at Vietnam National University of Agriculture (VNUA-2021/01) and at Kansas State University (IACUC#4845). All animal experiments were conducted strictly adhering to the IACUC protocols. Piglets (6–7 weeks old, female) that tested negative for ASFV and ASFV antibodies were obtained from clean pig farms and used for experiments in this study. Pigs were fed a standard commercial diet. In Vietnam, the pigs were housed in the Animal Biosafety Research Facility of the Faculty of Veterinary Medicine, Vietnam National University of Agriculture. In the United States, the pigs were housed under laboratory biosafety level III agriculture (BSL3-Ag) conditions at the Biosecurity Research Institute (BRI), Kansas State University (KSU).

### 2.3. Virus Passage, Titration, and Replication Test In Vitro

Serial passages of the ASFVs were conducted as previously described with slight modifications [39,40]. Briefly, monolayers of 3D4/21 cells at 90% confluency were infected with ASFVs at the multiplicity of infection (MOI) of 0.5. After a 2 h adsorption at 37 °C, the inocula were removed. Cells were rinsed twice with phosphate-buffered saline (PBS, pH 7.2, Thermo Scientific, Waltham, MA, USA), replaced with fresh culture media supplemented with 0.5% dimethyl sulfoxide (DMSO, Millipore Sigma, Lenexa, KS, USA), and incubated for 4 days. Culture supernatant was then harvested, titrated, and passaged onto fresh monolayers at an MOI of 0.5. Virus titers in the supernatants at each passage were titrated in PAMs. Briefly, PAMs were pre-seeded (80–100% confluent) and incubated with 10-fold dilutions of the harvested supernatants. For hemadsorption (HAD) testing, 2% porcine red blood cells were added after a 2 h incubation. After four days of culture, the presence of ASFV was assessed by HAD and cytopathic effects (CPEs) in tissue culture under an inverted microscope. HAD_50_ and TCID_50_ titers were calculated using the method of Reed and Muench [41].

For testing the in vitro replication characteristics of ASFVs, monolayers of PAMs and 3D4/21 cells at 90% confluency in 24-well culture plates were infected with the viruses at an MOI of 0.5. After a 2 h incubation, the inoculums were removed. Cells were washed and replaced with fresh culture media. Cultures (including cells and culture medium) were collected at 0, 24, 36, 48, 60, 72, 84, and 96 h post-infection (HPIs). The collected cultures were subjected to three freeze–thaw cycles. After centrifuging the cell debris, ASFV titers in the supernatant were tested and calculated as described above. All experiments were performed in duplicate.

### 2.4. ASFV Genome Next-Generation Sequencing

Next-generation sequencing of the ASFV genome was performed as described previously [40]. Briefly, ASFV DNA was extracted with the QIAamp DNA minikit (Qiagen, Hilden, Germany). The sequencing library was constructed following the manufacturer’s instruction of the NEBNext Ultra DNA Library Prep kit (New England Biolabs, Ipswich, MA, USA), and sequencing was performed with the Illumina NovaSeq 150PE sequencing platform (Illumina, San Diego, CA, USA). After sequencing, primer sequences were removed from raw Illumina reads using bbduk of the BBTools Packages (https://jgi.doe.gov/data-and-tools/bbtools/, last accessed 31 March 2024). QC reads were assembled *de novo* using SPAdes [42], polished using Pilon version 1.23 [43], and implemented in Unicycler [44]. All the contigs were subjected to BLASTN against the NCBI nucleotide database. Open reading frames (ORFs) were predicted using Prodigal [45] and annotated using Prokka 1.14.6 [46]. The single contig with BLASTN hit similarity to an ASFV was aligned with a number of reference genomes using MAFFT v7.450 [47]. The pairwise comparison of average nucleotide identity (ANI) between ASFV genomes was accomplished by ANI Calculator [48], which is available at https://www.ezbiocloud.net/tools/ani (last accessed 31 March 2024). Other tools for genomic visualization and classifying multigene family (MGF) proteins in ASFV were geneCo [49] and MGFC [50], respectively. All bioinformatic tools were run with default parameter settings. 

### 2.5. Safety, Efficacy, and Duration of Protection Testing of VNUA-ASFV-LAVL3 in Pigs

To evaluate the safety and efficacy of VNUA-ASFV-LAVL3, three groups of pigs (*n* = 5/group) were intramuscularly (i.m.) inoculated with 10^3^, 10^4^, and 10^5^ TCID_50_/dose of VNUA-ASFV-LAVL3, respectively. Two control groups (*n* = 3/group) received (i.m.) 10^2^ HAD_50_ of virulent VNUA-ASFV-L2 or DMEM. At 28 days post-inoculation (DPIs), all pigs were challenged (i.m.) with 10^3^ HAD_50_ of the wild-type VNUA-ASFV-05L1 strain for efficacy testing. Blood, oral fluids, rectal swabs, and serum samples were collected at various time points (0–28 DPIs and 0–28 days post-challenge, DPCs). Clinical signs (anorexia, depression, fever, purple skin discoloration, staggering gait, diarrhea, and cough), body temperature, and survival rate were monitored daily throughout the experiment by qualified personnel [26]. Dead pigs were necropsied for ASFV pathological lesions [2]. Tissue samples (1.5 g) from the inner part of the brain, tonsil, kidney, liver, spleen, lung, heart, lymph nodes, stomach, bladder, and bone marrow were aseptically collected for virus detection.

To assess the duration of protection afforded by VNUA-ASFV-LAVL3, two pig experiments (a one-month vaccination challenge and a two-month vaccination challenge) were conducted. In a one-month vaccination-challenge experiment, five pigs were vaccinated (i.m., once) with 10^4^ TCID_50_ VNUA-ASFV-LAVL3, three contact pigs (without vaccination) were added to test the shedding of the virus, and three control pigs were kept in a different room. After one month, all pigs were challenged with 10^3^ HAD_50_ of wild-type VNUA-ASFV-05L1 strain.

Similar to the one-month vaccination-challenge experiment, in the two-month vaccination-challenge experiment, five pigs were vaccinated (i.m., once) with 10^4^ TCID_50_ VNUA-ASFV-LAVL3, three contact pigs (without vaccination) were added to test the shedding of the virus, and three control pigs were kept in a different room. After two months, all pigs were challenged with 10^3^ HAD_50_ of the wild-type VNUA-ASFV-05L1 strain. Blood and serum samples were collected at 0, 30, and 60 days post-vaccination (DPVs), and at various DPCs (0, 3, 5, 7, 9, 11, 14, 17, 21, 25, and 28). The presence of clinical signs (anorexia, depression, fever, purple skin discoloration, staggering gait, diarrhea, and cough), body temperature, and survival rate were monitored daily throughout the experiment.

### 2.6. Quantitative PCR (qPCR) for ASFV

qPCR was used to detect ASFV DNA in oral fluid, rectal swabs, blood, and tissue samples collected from the experimental pigs. DNA extraction was performed using an automated King Fisher Duo Prime DNA/RNA extraction system (Thermo-Fisher Scientific, Waltham, MA, USA) with a MagMAX CORE nucleic acid purification kit (Life Sciences, New York, NY, USA) following the manufacturer’s instructions. Subsequently, ASFV DNA was quantified using the Platinum SuperMix-UDG kit (Invitrogen, Waltham, MA, USA) on the CFX Optus 96 Realtime PCR system (BioRad, Hercules, CA, USA). Specific primers and a probe targeting the ASFV *p72* gene, developed by Haines et al. [51], were employed. Samples with Ct values < 40 were considered positive for ASFV.

### 2.7. Detection of ASFV-Specific Antibody and Cellular Responses in Pigs

A commercial ASF-blocking ELISA kit (INGEZIM PPA COMPAC 11.PPA.k3, Ingenasa, Madrid, Spain) was used to detect anti-ASFV antibodies in serum samples. The assay was performed following the manufacturer’s instructions. Each sample’s competition percentage (S/N%) was calculated according to the manufacturer’s instructions, with interpretations as follows: ≥50% positive, 40–50% doubtful, and ≤40% negative.

Interferon-gamma (IFN-γ) production by peripheral blood mononuclear cells (PBMC) of pigs was assessed using an enzyme-linked immunospot assay (ELISPOT). Briefly, PBMCs were isolated from each pig using density-gradient centrifugation with 1.077 g/mL Ficoll-Paque PREMIUM density gradient media (Cytiva, Marlborough, MA, USA) in SepMate^TM^ tubes (STEMCELL Technologies Inc., Cambridge, MA, USA). PBMC suspensions (2 × 10^5^ cells/well) were added to MultiScreenHTS IP Filter Plates (Millipore Sigma, Lenexa, KS, USA) pre-coated with anti-pig IFN-γ antibodies (BD Biosciences, San Jose, CA, USA). PBMCs were incubated at 37 °C for 18 h with the following stimuli (100 μL/well): phorbol myristate acetate (PMA, 25 ng/mL)/ionomycin (2.5 μg/mL) (Millipore Sigma, Lenexa, KS, USA) combination as the positive control, VNUA-ASFV-05L1 (10^5^ HAD_50_/mL) as the re-stimulation agent, and complete culture media as the unstimulated control. After washing, biotinylated anti-pig IFN-γ (BD Biosciences, San Jose, CA, USA), HRP Streptavidin (BD Biosciences, San Jose, CA, USA), and fresh NovaRED Peroxidase Substrate (Vector Labs, Newark, CA, USA) were added as per the manufacturer’s instructions. The reaction was stopped by rinsing the plate with deionized water, and the number of IFN-γ spots was quantified using a CTL Spot Reader (CTL, New York, NY, USA).

### 2.8. Statistical Analysis

Statistical analysis was performed using GraphPad Prism 6.0 (San Diego, CA, USA). The data from assays for virus titration in cell cultures and blood samples in experimental pigs at different time points and efficacy studies were expressed as the mean log titer ± SD (standard deviation) for each group and analyzed with a Student’s *t*-test. The data for antibody and cellular responses were expressed as mean readings ± SD for each group. The significance of differences between the experimental groups was analyzed with an analysis of variance (ANOVA) followed by Turkey’s post-test. For all statistical analyses, *p* values less than 0.05 were considered statistically significant.

## 3. Results

### 3.1. VNUA-ASFV-LAVL3 Loses Hemadsorption Activity during Adaptation in 3D4/21 Cells

**Figure 1:**
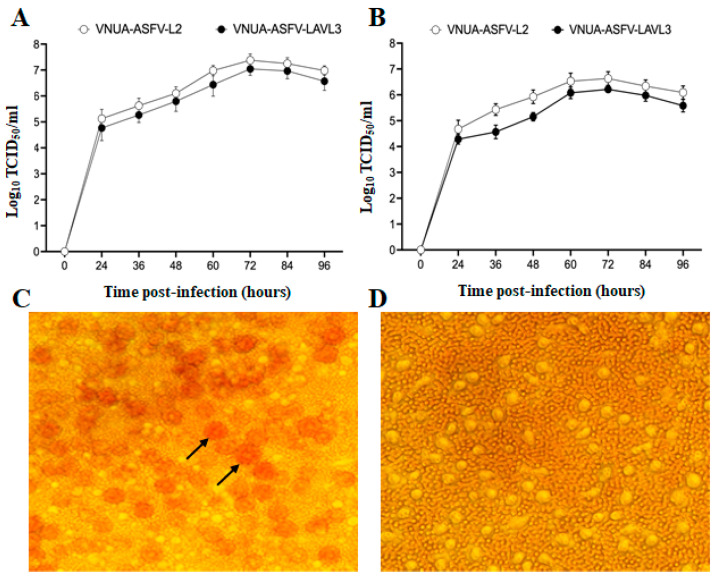
Growth characteristics and hemadsorption activity of VNUA-ASFV-LAVL3 (passage 50) and its parental virus VNUA-ASFV-L2. (**A**) Growth curves in PAMs. (**B**) Growth curves in 3D4/21 cells. (**C**) VNUA-ASFV-L2-infected 3D4/21 cells exhibit hemadsorption, 72 h post-infection (hpi), at magnification of 200×. (**D**) VNUA-ASFV-LAVL3-infected 3D4/21 cells, with no hemadsorption activity, 72 hpi, and magnification of 200×. Arrows indicate HAD rosettes.

VNUA-ASFV-LAVL3 was generated by serially passaging the virulent VNUA-ASFV-L2 strain (which had already undergone 70 passages in PAM cells) in 3D4/21 cells for a total of 50 times. We observed moderate viral growth in 3D4/21 cells, reaching titers of approximately 10^4^ to 10^5^ TCID_50_/mL by passage 12. The moderate-to-high titer (around 10^5^ TCID_50_/mL) was maintained throughout subsequent passages to passage 50, with the highest titer (10^6^ TCID_50_/mL) observed at 72 hpi. Compared to the parental VNUA-ASFV-L2 strain, VNUA-ASFV-LAVL3 exhibited comparable growth kinetics in both PAMs and 3D4/21 cells, with less than a 0.5 log10 difference in viral titers at each time point (Figure 1A,B). Notably, during adaptation and passaging in 3D4/21 cells, VNUA-ASFV-LAVL3 lost the hemadsorption activity characteristic of its parental virus, VNUA-ASFV-L2 (Figure 1C,D).

### 3.2. Genetic Characterization of VNUA-ASFV-LAVL3

**Figure 2:**
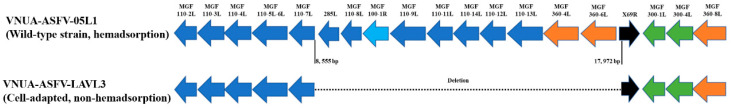
The graphical panel highlights the deletion of known genes in the MGF regions of the VNUA-ASFV-LAVL3 strain. This panel displays a schematic representation of the ASFV genome organization. Different colors represent major ORF categories. The dashed lines highlight the deleted region in the VNUA-ASFV-LAVL3 strain.

Whole-genome sequencing revealed that the genome of VNUA-ASFV-LAVL3 comprises 179,678 bp with a GC content of 36.34%. Comparative analysis with the wild-type VNUA-ASFV-05L1 (GenBank accession number MW465755.1) identified a notable deletion encompassing a region spanning from nucleotides (nt) of 8555 bp to 17972 in the genome of VNUA-ASFV-05L1. This deletion resulted in the loss of 10 known genes (*285L*, *MGF 110-8L*, *MGF 100-1R*, *MGF 110-9L*, *MGF 110-11L*, *MGF 110-14L*, *MGF 110-12L*, *MGF 110-13L*, *MGF360-4L*, and *MGF360-6L*), a partial sequence loss of the *X69R* gene, and the deletion of 11 uncharacterized sequences (Figure 2). 

Additionally, insertions and mutations leading to amino acid substitutions or protein disruptions were discovered in the genome of VNUA-ASFV-LAVL3 (Table 1). Of particular interest were three alterations within the *EP402R* (*CD2v*) gene: a single nucleotide substitution (72222 thymine to cytosine) and two single-nucleotide deletions (72844 thymine and 72965 cytosine). These changes potentially disrupt the CD2v protein, which plays a crucial role in the hemadsorption of swine red blood cells (RBCs) [52].

### 3.3. VNUA-ASFV-LAVL3 Exhibits High Safety in Pigs

**Figure 3:**
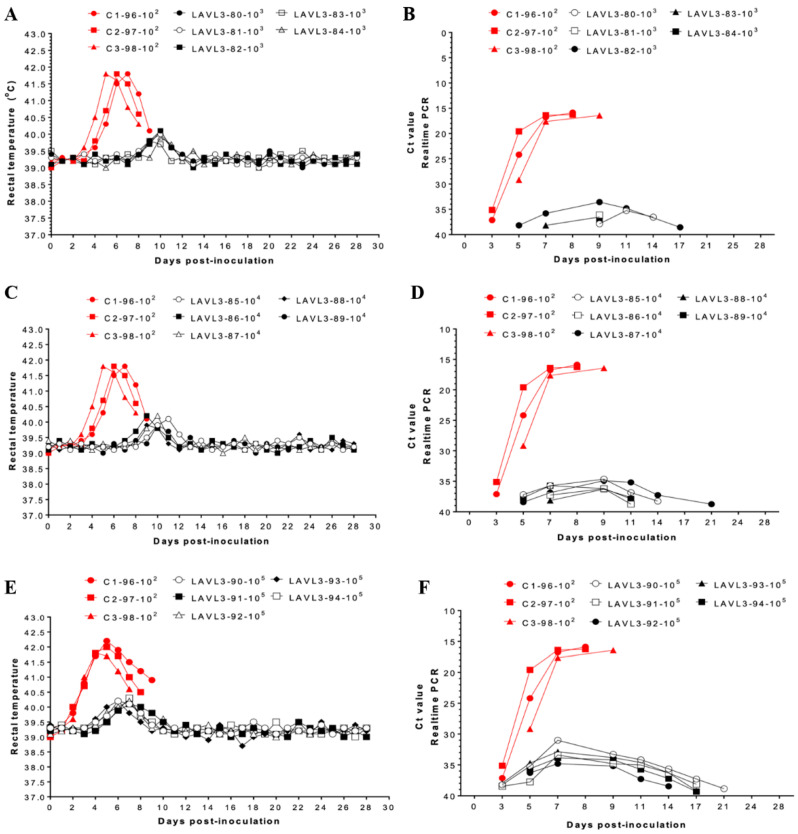
Rectal temperature and viremia in pigs inoculated with VNUA-ASFV-LAVL3 or parental VNUA-ASFV-L2 virus. Daily rectal temperature of the control pigs and the pigs inoculated with (**A**) 10^3^ TCID_50_, (**C**) 10^4^ TCID_50_, and (**E**) 10^5^ TCID_50_ of VNUA-ASFV-LAVL3. Viremia of the control pigs and the pigs inoculated with (**B**) 10^3^ TCID_50_, (**D**) 10^4^ TCID_50_, and (**F**) 10^5^ TCID_50_ of VNUA-ASFV-LAVL3. Legend: “C”, control pig; “LAVL3”, VNUA-ASFV-LAVL3; “number in the middle”, individual pig identification; “numbers at the end”, inoculated virus dose.

Pigs inoculated with 10^3^, 10^4^, and 10^5^ TCID_50_ of VNUA-ASFV-LAVL3 exhibited a temporary low fever (<40.6 °C) between 9–11 DPIs (peak at 10 DPIs) (Figure 3A), 8–11 DPIs (peak at 10 DPIs) (Figure 3C), and 5–9 DPIs (peak at 7 DPIs) (Figure 3E), respectively. Apart from this, vaccinated pigs maintained normal body temperature throughout the 28-day observation period and showed no obvious ASFV-specific clinical signs. In contrast, control pigs inoculated with the parental VNUA-ASFV-L2 virus developed fever as early as 3 DPIs (peak at 5 DPIs with 41–41.8 °C) (Figure 3A,C,E) followed by severe ASF clinical signs (anorexia, coughing, depression, purple skin discoloration, respiratory symptoms, and diarrhea) and died within 8–9 DPIs (Figure 3A,C,E).

VNUA-ASFV-LAVL3 vaccinated pigs showed very low viremia, with Ct values ranging from 34.62 to 37.51 (low dose) between 5 and17 DPIs and from 31.52 to 35.17 (high dose) between 3 and 21 DPIs. This viremia rapidly declined, becoming almost undetectable by 17 DPIs (Figure 3B,D,F). On the other hand, control pigs inoculated with the parental VNUA-ASFV-L2 virus reached peak viremia (Ct value~15) at 7 DPIs, remaining high until death by 8–9 DPIs (Figure 3B,D,F). Additionally, ASFV was undetectable in oral fluid or rectal swabs from vaccinated pigs throughout the 28-day observation period (Supplementary Table S1).

### 3.4. Vaccination with VNUA-ASFV-LAVL3 Confers Full Protection in Pigs

**Figure 4:**
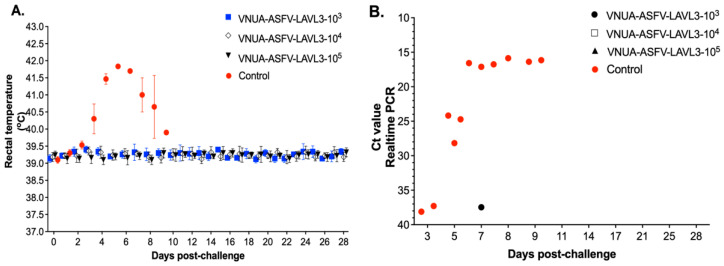
Rectal temperature and viremia in pigs following challenge with virulent wild-type VNUA-ASFV-05L1 strain (genotype II). (**A**) Daily rectal temperature of the vaccinated and nonvaccinated control pigs after challenged with 10^3^ HAD_50_ of VNUA-ASFV-05L1 strain. (**B**) Viremia of vaccinated and non-vaccinated control pigs after challenged with 10^3^ HAD_50_ of VNUA-ASFV-05L1.

To evaluate the potential of VNUA-ASFV-LAVL3 as a live-attenuated virus vaccine, pigs vaccinated with different doses of VNUA-ASFV-LAVL3 were challenged intramuscularly with 10^3^ HAD_50_ of the highly virulent wild-type VNUA-ASFV-05L1 strain at 28 DPIs. All the vaccinated groups maintained normal rectal temperatures after the challenge, unlike control pigs whose temperatures rapidly increased to 41.8 °C at 5 DPCs (Figure 4A). The challenge virus was almost undetectable in blood samples of all vaccinated groups. Only one pig in the 10^3^ TCID_50_ dose group showed low viral titer (a Ct value of 37.84) at 7 DPCs (Figure 4B). All vaccinated pigs remained healthy and fully protected against the virulent challenge, achieving 100% survival throughout the 28-day DPC observation. In contrast, the challenge virus was detected in control pigs as early as 3–5 DPCs, rapidly increasing by 5–7 DPCs (Figure 4B). They developed typical ASF clinical signs at 5–9 DPCs and succumbed to death by 8–9 DPCs.

### 3.5. VNUA-ASFV-LAVL3 Vaccination Prevents Replication of Wild-Type ASFV and Protects against Pathological Lesions in Pigs

To further validate the protective efficacy conferred by VNUA-ASFV-LAVL3 in pigs, we investigated the presence of a virulent challenge strain and its associated pathological lesions in various organs of vaccinated pigs at 28 DPCs and control pigs at 8–9 DPCs. At 28 DPCs, organs (brain, heart, lung, liver, stomach, spleen, kidney, bladder, tonsil, lymph node, and bone marrow) collected from control pigs displayed high ASFV titers as determined by qPCR analysis (Table 2). In contrast, pigs vaccinated with VNUA-ASFV-LAVL3 at various doses (10^3^–10^5^ TCID_50_) showed undetectable levels of ASFV in all examined organs at 28 DPCs (Table 2). These data suggest that VNUA-ASFV-LAVL3 vaccination effectively prevents both viral replication and the development of pathological lesions in pigs challenged with the virulent ASFV strain.

**Figure 5:**
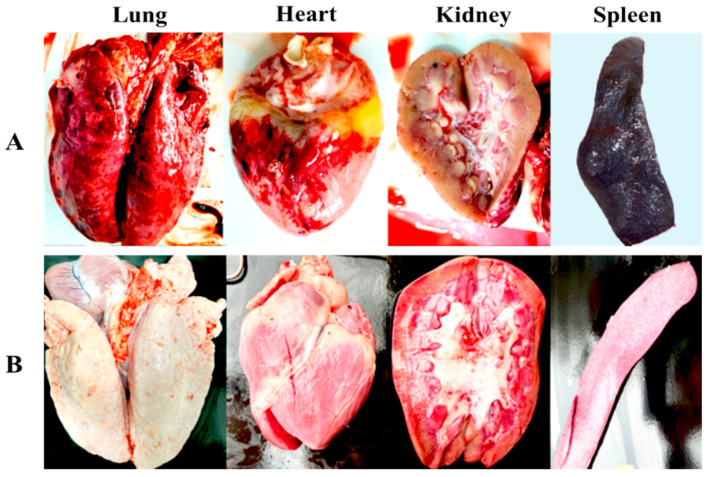
Pathological lesion findings in organs of the vaccinated and non-vaccinated pigs post-challenge at necropsy. (**A**) Organs from the unvaccinated control pigs at 9 DPCs. (**B**) Organs from the pigs vaccinated with the VNUA-ASFV-LAVL3 strain at 28 DPCs.

Post-mortem examination of the non-immunized control pigs challenged with the wild-type VNUA-ASFV-05L1 revealed severe gross lesions and pathological signs of acute ASF, as previously described [2,53]. These pigs displayed extensive macroscopic lesions, including necrosis and hemorrhage in various organs: mandibular lymph nodes, lungs, mesentery, spleen, renal cortex, pericardium, and myocardium. Additionally, the non-immunized pigs exhibited dark and enlarged spleens, swollen livers and gallbladders, and interstitial pulmonary edema (Figure 5A). Conversely, the pigs vaccinated with VNUA-ASFV-LAVL3 showed no clinical signs, pathological lesions, or abnormalities in any examined organs at necropsy (Figure 5B).

### 3.6. Pigs with VNUA-ASFV-LAVL3 Induce Robust ASFV-Specific Humoral and Cellular Immune Responses

**Figure 6:**
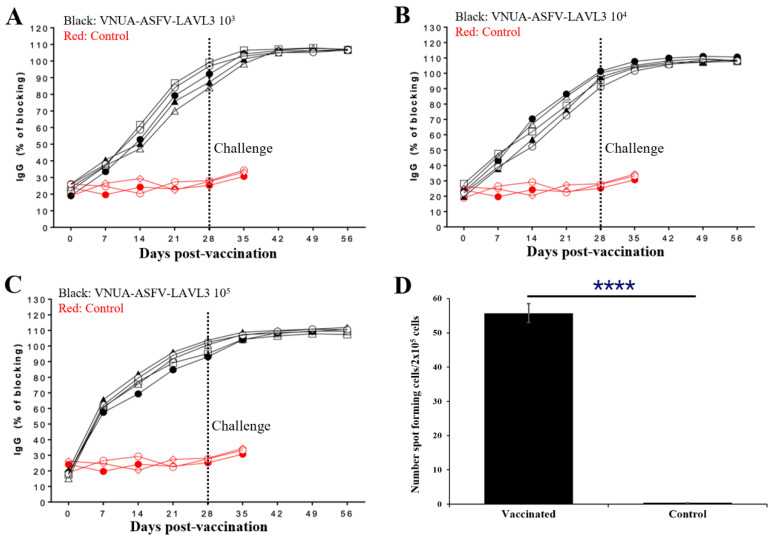
Humoral and cellular immune responses of the VNUA-ASFV-LAVL3-vaccinated pigs and the non-vaccinated control pigs. (**A**) ASFV-specific antibodies in the serum samples of pigs vaccinated with 10^3^ TCID_50_ of VNUA-ASFV-LAVL3 and control pigs. (**B**) ASFV-specific antibodies in serum samples of the pigs vaccinated with 10^4^ TCID_50_ of VNUA-ASFV-LAVL3 and the control pigs. (**C**) ASFV-specific antibodies in the serum samples of the pigs vaccinated with 10^5^ TCID_50_ of VNUA-ASFV-LAVL3 and the control pigs. (**D**) ELISPOT testing of ASFV-specific IFN-γ-producing PBMCs at 28 DPVs, **** *p* < 0.0001.

Pigs vaccinated with a dose of 10^3^, 10^4^, and 10^5^ TCID_50_ of the VNUA-ASFV-LAVL3 strain developed anti-ASFV antibodies as early as 7 days post-vaccination (DPVs). At 28 DPVs (0 DPCs), the blocking percentage (%) of ASFV-specific antibodies reached approximately 83.6–103.7%, whereas no ASFV-specific antibodies were detected in the control animals prior to the challenge (Figure 6A–C). Following the challenge with the wild-type VNUA-ASFV-05L1, the antibody levels peaked at 7 DPCs (35 DPIs), and remained consistent up to 28 DPCs (56 DPIs) at the end of the experiment (Figure 6A–C). ELIspot assay results showed that positive controls exhibited spot counts exceeding 200. The PBMCs of VNUA-ASFV-LAVL3-vaccinated pigs produced IFN-γ after stimulation with the wild-type VNUA-ASFV-05L1. The numbers of spot-forming cells from the VNUA-ASFV-LAVL3-vaccinated pigs were significantly (*p* < 0.0001) higher than the cells from the non-vaccinated control pigs (Figure 6D).

### 3.7. Pigs Can Be Protected against Wild-Type ASFV Challenge for Up to 2 Months after a Single Vaccination with VNUA-ASFV-LAVL3

We evaluated the ability to confer long-term protection (1 month and 2 months) of the VNUA-ASFV-LAVL3 using a single dose (10^4^ TCID_50_) in pigs. As expected, all the vaccinated pigs developed consistent ASFV-specific antibody responses with sustained immune responses up to 2 months post-vaccination. All the contact pigs were negative for ASFV-specific antibody responses and ASFV until the moment of challenge, indicating no ASFV infection (Supplementary Table S2). After the challenge, two out of five vaccinated pigs in the 2-month vaccination group showed low levels of ASFV (real-time PCR Ct value ranging from 37.51 to 36.18) at 7 and 9 DPCs, and showed negative at later time points post-challenge. All the vaccinated pigs survived, remained healthy, and had no clinical signs of ASFV infection after challenging with wild-type VNUA-ASFV-05L1 (Table 3). The control pigs and contact pigs developed a fever at 3 DPCs. Subsequently, clinical signs (anorexia, coughing, depression, purple skin discoloration, severe respiratory symptoms, and diarrhea) of acute ASF were observed. All the control pigs and contact pigs became viremic at 5–7 DPCs and were euthanized at 8–11 DPCs (Table 3).

## 4. Discussion

Live-attenuated vaccines, generated by serial passaging of a virus in cultured cells, have proven highly effective in preventing numerous viral diseases, including smallpox, polio, measles, mumps, and yellow fever. These LAVs elicit robust cellular and humoral immune responses, often providing lifelong protection with minimal dosing (one or two doses) [54,55]. Carefully controlling passage numbers during cell culture is crucial to balance viral replication and immune induction for developing cell-passaged LAVs. Excessive passaging can weaken the vaccine by eliminating essential protective genes [23]. Through careful cell culture and controlled passage times, we have successfully generated an HAD-positive ASF LAV candidate [26]. Here, we report a non-HAD ASF LAV candidate, VNUA-ASFV-LAVL3, developed using the same approach. The cell-adapted VNUA-ASFV-LAVL3 can efficiently infect and replicate in 3D4/21 cells with 0.5% DMSO in the culture medium (Figure 1B). Earlier studies highlighted the potential of non-HAD ASFV strains for LAV development. The naturally attenuated non-HAD ASFV/L60 and Lv17/WB/Rie1 strains displayed significantly lower virulence than their parental virulent HAD strains [55,56]. Consistent with these findings, the non-HAD VNUA-ASFV-LAVL3 exhibited highly attenuated properties, showing promise for inducing protective immunity against wild-type genotype II ASFV in pigs (Figure 3, Figure 4 and Figure 5). 

The *EP402R* gene, encoding the CD2v protein, is responsible for the ASFV distinctive HAD phenomenon in ASFV [55,56]. We identified a single-nucleotide substitution (72222 thymine to cytosine) and two single-nucleotide deletions (72844 thymine and 72965 cytosine) in the *EP402R* gene of VNUA-ASFV-LAVL3 (Table 1), leading to a frameshift variant in the CD2v protein, shedding light on the genetic basis for the loss of hemadsorption with the VNUA-ASFV-LAVL3 strain. Additionally, the MGF region in ASFV plays a crucial role in viral virulence, and deletions within this region are associated with attenuated phenotypes [26,35,36,37,38]. Full-genome sequencing revealed deletions of 10 genes (*285L*, *MGF 110-8L*, *MGF 100-1R*, *MGF 110-9L*, *MGF 110-11L*, *MGF 110-14L*, *MGF 110-12L*, *MGF 110-13L*, *MGF360-4L*, and *MGF360-6L*) and a partial deletion in the *X69R* gene within the MGF region of VNUA-ASFV-LAVL3 (Table 1), potentially contributing to its attenuated phenotype in pigs. Notably, VNUA-ASFV-LAVL3 exhibited comparable growth kinetics to the parental strain in 3D4/21 cells, indicating that the observed mutations and deletions are not associated with impaired viral replication. 

A coordinated host response involving both cellular and humoral immunity is essential for combating ASFV infection [57]. Cellular immunity, particularly T cell-mediated responses, plays a pivotal role in controlling viral replication by targeting infected cells [58]. ELISPOT assays demonstrated that VNUA-ASFV-LAVL3 induced robust cellular immune responses (ASFV-specific IFN-γ) in pigs. While the precise role of antibodies in ASFV protection remains unclear, their presence is considered crucial for defense. Pigs vaccinated with VNUA-ASFV-LAVL3 at doses of 10^3^, 10^4^, and 10^5^ TCID50 developed anti-ASFV antibodies as early as 7 DPVs, consistent with previous findings of antibody detection within 7–14 days post-infection in pigs infected with low or moderately virulent ASFV strains [59,60,61]. However, the exact mechanisms of how cellular and humoral immune responses work together in combating ASFV require further investigation.

A potential concern with LAVs is the risk of shedding and potential transmission or reversion to virulence [2,21]. In our study, no transmission of VNUA-ASFV-LAVL3 from vaccinated pigs to unvaccinated contact pigs was observed, even when challenged 1 or 2 months post-vaccination. All contact pigs remained negative for ASFV-specific antibodies until the challenge. Furthermore, VNUA-ASFV-LAVL3 DNA was undetectable in the organs of vaccinated pigs (Table 2). Notably, a single dose (10^4^ TCID_50_) of VNUA-ASFV-LAVL3 effectively protected pigs against wild-type ASFV challenge for up to 2 months (Table 3). While this study focused on 6–7-week-old piglets, the results strongly support a further investigation of VNUA-ASFV-LAVL3 as a potential broad-spectrum ASFV vaccine. Future studies will assess the vaccine’s efficacy in diverse swine populations, including piglets, sows, pregnant sows, and boars. Additionally, we will evaluate its protective capacity against emerging ASFV variants, such as the increasingly prevalent genotype I and II recombinant strains in Asia [62].

## 5. Conclusions

This study successfully developed VNUA-ASFV-LAVL3, a highly safe and efficacious vaccine candidate against genotype II ASFV, using cell passage technology. The vaccine candidate efficiently replicates in the commercially available swine macrophage cell line 3D4/21, eliciting robust humoral and cellular immune responses. Importantly, VNUA-ASFV-LAVL3 provided long-term protection against challenges with a highly virulent genotype II ASFV strain. 

## Figures and Tables

**Table 1:** Mutations of the VNUA-ASFV-LAVL3 genome using the reverse-complement sequence of the wild-type VNUA-ASFV-05L1 (GenBank accession number MW465755.1) as a reference.

Genome Position	Mutation Type	Gene	Nucleotide Change	Amino Acid Change	Protein Change
6343 to 6344	Insertion	Uncharacterized	61nt	Uncharacterized	Uncharacterized
6366	Substitution	Uncharacterized	T to A	Uncharacterized	Uncharacterized
6371	Substitution	Uncharacterized	G to A	Uncharacterized	Uncharacterized
8555 to 8718	Deletion	Uncharacterized	Deletion	Deletion	Deletion
8719 to 9003	Deletion	*285L*	Deletion	Deletion	Deletion
9004 to 9131	Deletion	Uncharacterized	Deletion	Deletion	Deletion
9132 to 9515	Deletion	*MGF110-8L*	Deletion	Deletion	Deletion
9516 to 9732	Deletion	Uncharacterized	Deletion	Deletion	Deletion
9733 to 10107	Deletion	*MGF_100-1R*	Deletion	Deletion	Deletion
10108 to 10265	Deletion	Uncharacterized	Deletion	Deletion	Deletion
10266 to 11138	Deletion	*MGF_100-9L*	Deletion	Deletion	Deletion
11139 to 11428	Deletion	Uncharacterized	Deletion	Deletion	Deletion
11429 to 11788	Deletion	*MGF110-11L*	Deletion	Deletion	Deletion
11789 to 11877	Deletion	Uncharacterized	Deletion	Deletion	Deletion
11878 to 12243	Deletion	*MGF110-14L*	Deletion	Deletion	Deletion
12244 to 12432	Deletion	Uncharacterized	Deletion	Deletion	Deletion
12433 to 12792	Deletion	*MGF110-12L*	Deletion	Deletion	Deletion
12793 to 12878	Deletion	Uncharacterized	Deletion	Deletion	Deletion
12879 to 13702	Deletion	*MGF110-13L*	Deletion	Deletion	Deletion
13703 to 13882	Deletion	Uncharacterized	Deletion	Deletion	Deletion
13883 to 15046	Deletion	*MGF360-4L*	Deletion	Deletion	Deletion
15047 to 15862	Deletion	Uncharacterized	Deletion	Deletion	Deletion
15863 to 16990	Deletion	*MGF360-6L*	Deletion	Deletion	Deletion
16991 to 17868	Deletion	Uncharacterized	Deletion	Deletion	Deletion
17869 to 17972	Deletion	*X69R*	104 nt	34 aa	Shorter protein
20076	Deletion	*MGF_300-2R*	C	D to I	Disruption
20188	Substitution	*MGF_300-2R*	T to C	S to N	Disruption
23952–23962	Deletion	Uncharacterized	11 nt	Uncharacterized	Uncharacterized
24103	Substitution	*MGF_360-10L*	T to C	D to Q	Disruption
24752	Deletion	*MGF_360-10L*	A	D to E	Disruption
28221–28324	Deletion	*MGF_360-12L*	104 nt	34aa	Shorter protein
30324	Deletion	Uncharacterized	A	Uncharacterized	Uncharacterized
30871	Substitution	*MGF_360-14L*	G to A	H to A	Abnormal protein
30876–30877	Substitution	*MGF_360-14L*	TC to AT	I to S	Abnormal protein
30879	Substitution	*MGF_360-14L*	C to T	D to V	Abnormal protein
30882–30915	Deletion	*MGF_360-14L*	34 nt	11 aa	Abnormal protein
30918	Substitution	*MGF_360-14L*	T to A	N to Y	Abnormal protein
30923	Substitution	*MGF_360-14L*	T to A	Y to F	Abnormal protein
30930	Substitution	*MGF_360-14L*	T to A	S to C	Abnormal protein
30938	Substitution	*MGF_360-14L*	A to C	V to G	Abnormal protein
33291	Deletion	*MGF_505-2R*	A	K to T	Shorter protein
36919	Substitution	*MGF_505-5R*	T to C	Y to N	Substitution
40351	Substitution	*MGF_505-7R*	T to G	V to G	Substitution
41114	Deletion	Uncharacterized	T	Uncharacterized	Uncharacterized
42931–42947	Deletion	Uncharacterized	17 nt	Uncharacterized	Uncharacterized
43100–43133	Deletion	Uncharacterized	34 nt	Uncharacterized	Uncharacterized
46011	Deletion	*A104R*	A	K to S	Disruption
53169	Deletion	Uncharacterized	T	Uncharacterized	Uncharacterized
58635	Substitution	*F1055L*	T to C	I to W	Disruption
58913	Substitution	*F1055L*	T to G	H to L	Disruption
60945–61020	Deletion	*F1055L*	76 nt	25 aa	Disruption
61819	Substitution	Uncharacterized	T to C	Uncharacterized	Uncharacterized
72222	Substitution	*EP402R*	T to C	C to R	Disruption
72824	Deletion	*EP402R*	T	R to E	Disruption
72965	Deletion	*EP402R*	C	P to T	Disruption
75240–75320	Deletion	*M1249L*	81 nt	27 aa	Abnormal protein
77036	Substitution	*M1249L*	T to C	M to V	Abnormal protein
78038	Substitution	*M1249L*	T to G	N to H	Abnormal protein
85588	Substitution	*C475L*	T to G	R to S	Substitution
93313	Substitution	*B962L*	A to G	Y to H	Substitution
102005	Substitution	*B385R*	A to G	T to A	Substitution
105743–105744	Insertion	*B407L*	40nt	13 aa	Disruption
105777	Substitution	*B407L*	T to C	S to G	Disruption
107968–107969	Insertion	*G1340L*	19 nt	6 aa	Abnormal protein
109399	Substitution	*G1340L*	A to C	V to G	Abnormal protein
114357–114561	Deletion	*G1211R*	205 nt	68 aa	Disruption
120939–121034	Deletion	*CP2475L*	96 nt	32 aa	Abnormal protein
122809	Substitution	*CP2475L*	T to C	N to S	Abnormal protein
124660	Substitution	*CP530R*	A to G	N to D	Substitution
130367–130446	Deletion	*NP1450L*	80 nt	26 aa	Disruption
131201–131500	Deletion	*NP1450L*	300 nt	100 aa	Disruption
131815	Substitution	*NP1450L*	A to G	I to T	Disruption
136825–136841	Deletion	*D250R*	17 nt	6 aa	Shorter protein
141015	Substitution	*D1133L*	T to C	E to A	Disruption
141043	Substitution	*D1133L*	T to G	T to S	Disruption
141285–141420	Deletion	*D1133L*	136 nt	48 aa	Disruption
145156–145171	Deletion	*S183L*	16 nt	5 aa	Disruption
145490	Substitution	*S273R*	C to A	S to S	None
146353–146375	Deletion	*P1192R*	23 nt	7 aa	Disruption
153308	Substitution	*H233R*	T to C	V to A	Disruption
153598–153665	Deletion	*H233R*	68	22 aa	Disruption
154180	Substitution	*H240R*	C to T	L to F	Substitution
166366	Substitution	*E248R*	A to C	P to P	None
173569–173592	Deletion	*I177L*	24	8 aa	Abnormal protein
174320–174320	Deletion	Uncharacterized	A	Uncharacterized	Uncharacterized
182192	Deletion	*MGF 360-18R*	A	E to N	Disruption
182665	Substitution	*MGF 360-18R*	T to C	L to N	Disruption

**Table 2:** ASFV detection in organs of VNUA-ASFV-LAVL3 vaccinated pigs and control pigs.

Organs	Quantitative ASFV Real-Time PCR																	
	10^3^ TCID_50_ Vaccinated (Pig Number)					10^4^ TCID_50_ Vaccinated (Pig Number)					10^5^ TCID_50_ Vaccinated (Pig Number)					Control (Pig Number)		
	80	81	82	83	84	85	86	87	88	89	90	91	92	93	94	96	97	98
Brain	-	-	-	-	-	-	-	-	-	-	-	-	-	-	-	+	+	+
Heart	-	-	-	-	-	-	-	-	-	-	-	-	-	-	-	+	+	+
Lung	-	-	-	-	-	-	-	-	-	-	-	-	-	-	-	+	+	+
Liver	-	-	-	-	-	-	-	-	-	-	-	-	-	-	-	+	+	+
Stomach	-	-	-	-	-	-	-	-	-	-	-	-	-	-	-	+	+	+
Spleen	-	-	-	-	-	-	-	-	-	-	-	-	-	-	-	+	+	+
Kidney	-	-	-	-	-	-	-	-	-	-	-	-	-	-	-	+	+	+
Bladder	-	-	-	-	-	-	-	-	-	-	-	-	-	-	-	+	+	+
Tonsil	-	-	-	-	-	-	-	-	-	-	-	-	-	-	-	+	+	+
ILN	-	-	-	-	-	-	-	-	-	-	-	-	-	-	-	+	+	+
MLN	-	-	-	-	-	-	-	-	-	-	-	-	-	-	-	+	+	+
SLN	-	-	-	-	-	-	-	-	-	-	-	-	-	-	-	+	+	+
BM	-	-	-	-	-	-	-	-	-	-	-	-	-	-	-	+	+	+

Note: ILN: inguinal lymph node; MLN: mesenteric lymph node; SLN: submaxillary lymph node; BM: bone-marrow; “-”: negative results for ASFV DNA with qPCR; “+”: positive results for ASFV DNA with real-time PCR.

**Table 3:** Long-term protection conferred by a single vaccination of VNUA-ASFV-LAVL3 in experimental pigs.

Group	No. of Pigs	Vaccination	Wild-Type ASFV Challenge at	Mean (±SD) Time to Death (Days)	Mean (±SD) Fever Duration (Days)	Survival/Protection Rate (%)	Clinical Signs
A	5	10^4^ VNUA-ASFV-LAVL3	1-month post-vaccination	0	0	5/100	Healthy
	3	No vaccination, contact		10.3 (±0.57)	5.6 (±0.57)	0/0	High fever, ASF clinical signs
B	3	No vaccination		8.3 (±0.57)	4.3 (±0.57)	0/0	High fever, ASF clinical signs
C	5	10^4^ VNUA-ASFV-LAVL3	2-month post-vaccination	0	0	5/100	Healthy
	3	No vaccination, contact		10.3 (±0.57)	5.6 (±0.57)	0/0	High fever, ASF clinical signs
D	3	No vaccination		8.6 (±0.57)	4.6 (±0.57)	0/0	High fever, ASF clinical signs

## Data Availability

The datasets for the current study are available from the corresponding author upon reasonable request.

## Supplementary Materials

The following supporting information can be downloaded at: https://www.mdpi.com/article/10.3390/v16081326/s1, Table S1: ASFV detection in oral fluid and rectal swabs from vaccinated pigs by real-time PCR, 0-28 DPI; Table S2: Detection of ASFV-specific antibodies using the commercial ASF blocking ELISA kit (INGEZIM PPA COMPAC 11.PPA.k3, Ingenasa, Madrid, Spain).
