# Supplementary material for: A Non-Hemadsorbing Live-Attenuated Virus Vaccine Candidate Protects Pigs against the Contemporary Pandemic Genotype II African Swine Fever Virus
Source: Viruses. 2024 Aug 19;16(8):1326. doi: 10.3390/v16081326 (PMC11359042; doi:10.3390/v16081326)
Supplement: Supplementary file 1 [file viruses-16-01326-s001.zip › viruses-3105500-supplementary.pdf]

Supplementary Table S1: ASFV detection in oral fluid and rectal swabs from vaccinated pigs by real-time PCR, 0-28 DPI.

Samples	ASFV Real-time PCR														
	10^3 $TCID_{50}$ vaccinated 0-28 DPI (pig number)					10^4 $TCID_{50}$ vaccinated 0-28 DPI (pig number)					10^5 $TCID_{50}$ vaccinated 0-28 DPI (pig number)				
	80	81	82	83	84	85	86	87	88	89	90	91	92	93	94
**Oral fluid**	-	-	-	-	-	-	-	-	-	-	-	-	-	-	-
**Rectal swabs**	-	-	-	-	-	-	-	-	-	-	-	-	-	-	-

Note: "-" under the detection threshhold of ASFV Real-time PCR.

Supplementary Table S2: Detection of ASFV-specific antibodies using the commercial ASF blocking ELISA kit (INGEZIM PPA COMPAC 11.PPA.k3, Ingenasa, Madrid, Spain).

Group	No. of pigs	Vaccination	ASFV-specific antibodies Blocking percentage (%)	Clinical signs
1-month vaccination, 0 DPC	5	10^4 VNUA-ASFV-LAVL3	>83%	Healthy
	3	No vaccination, contact	<40%	Healthy
	3	No vaccination	<40%	Healthy
2-months vaccination, 0 DPC	5	10^4 VNUA-ASFV-LAVL3	>90%	Healthy
	3	No vaccination, contact	<40%	Healthy
	3	No vaccination	<40%	Healthy

Note: Each sample's competition percentage (S/N%) was calculated according to the manufacturer's instructions, with enterpretations as follows: ≥ 50% positive, 40-50% doubtful, and ≤40% negative.
